# Monoclonal Antibodies in Dermatooncology—State of the Art and Future Perspectives

**DOI:** 10.3390/cancers11101420

**Published:** 2019-09-24

**Authors:** Malgorzata Bobrowicz, Radoslaw Zagozdzon, Joanna Domagala, Roberta Vasconcelos-Berg, Emmanuella Guenova, Magdalena Winiarska

**Affiliations:** 1 Department of Immunology, Medical University of Warsaw, 02-097 Warsaw, Poland; malgorzata.bobrowicz@wum.edu.pl (M.B.); j.b.stachura@gmail.com (J.D.); 2 Department of Clinical Immunology, Medical University of Warsaw, 02-006 Warsaw, Poland; Radoslaw.zagozdzon@wum.edu.pl; 3 Department of Immunology, Transplantology and Internal Diseases, Medical University of Warsaw, 02-006 Warsaw, Poland; 4 Postgraduate School of Molecular Medicine, 02-091 Warsaw, Poland; 5 Department of Dermatology, University Hospital Basel, University of Basel, 4031 Basel, Switzerland; robertadermatousp@gmail.com; 6 Department of Dermatology, University Hospital Zurich, University of Zurich, 8091 Zurich, Switzerland; 7 Department of Dermatology, University of Lausanne, 1011 Lausanne, Switzerland

**Keywords:** dermatooncology, immune checkpoints, immunotherapy, monoclonal antibodies

## Abstract

Monoclonal antibodies (mAbs) targeting specific proteins are currently the most popular form of immunotherapy used in the treatment of cancer and other non-malignant diseases. Since the first approval of anti-CD20 mAb rituximab in 1997 for the treatment of B-cell malignancies, the market is continuously booming and the clinically used mAbs have undergone a remarkable evolution. Novel molecular targets are constantly emerging and the development of genetic engineering have facilitated the introduction of modified mAbs with improved safety and increased capabilities to activate the effector mechanisms of the immune system. Next to their remarkable success in hematooncology, mAbs have also an already established role in the treatment of solid malignancies. The recent development of mAbs targeting the immune checkpoints has opened new avenues for the use of this form of immunotherapy, also in the immune-rich milieu of the skin. In this review we aim at presenting a comprehensive view of mAbs’ application in the modern treatment of skin cancer. We present the characteristics and efficacy of mAbs currently used in dermatooncology and summarize the recent clinical trials in the field. We discuss the side effects and strategies for their managing.

## 1. Monoclonal Antibodies

Monoclonal antibodies (mAbs) targeting specific proteins are the most popular form of immunotherapy used in the treatment of cancer and non-malignant diseases. Since the first approval of anti-CD20 mAb rituximab in 1997 for the treatment of B-cell malignancies, the clinically used mAbs have undergone a remarkable evolution. Novel molecular targets are constantly emerging and the development of genetic engineering has facilitated the introduction of mAbs with modified structures. Recent advances in the field of monoclonal antibodies served to improve their safety, decrease immunogenicity and increase capabilities to activate the effector mechanisms of the immune system. Monoclonal antibodies are a booming market currently [1], making up a third of all new medicines introduced worldwide. The success of mAbs is remarkable in recent years with a record number of 12 novel antibodies registered in 2018 to treat a wide variety of diseases [2]. 

The concept of using antibodies to selectively target tumors has undergone a considerable evolution (excellently reviewed in [3]), since it has been proposed by Paul Ehrlich over a century ago. An update on the technological advances and novel registrations is published yearly by Hélène Kaplon and Janice Reichert in the mAbs journal [2]. 

The mechanisms of action of the currently used mAbs in dermatooncology can be generalized in five main directions: (1) The direct inhibition of oncogenic pathways with subsequent effects on cell growth and apoptosis e.g., targeting the epidermal growth factor receptor (EGFR), (2) the blockade of the formation of new blood vessels e.g., targeting vascular endothelial growth factor VEGF, (3) the opsonization of the target cells for its destruction utilizing the mechanisms of immune response e.g., targeting CD20, (4) the delivery of cytotoxic drugs to kill tumor cells e.g., mAb-cytotoxin conjugates such as anti-CD30 brentuximab vedotin and finally, (5) the activation of the impaired/exhausted immune response i.e., targeting negative immune regulators also known as immune checkpoints (ICs). Currently, particularly this last trend, awarded the 2018 Nobel Prize in Physiology or Medicine to Allison and Honjo, is of particular interest in the therapy of cancer.

Here we aim at presenting the established position, recent advances and perspectives in the treatment of cutaneous neoplasms with monoclonal antibodies.

### Immune Checkpoints

The operational principle of the adoptive part of the immune system relies on two main functions: (1) Distinguishing self from non-self and (2) distinguishing safe from dangerous. This allows the immune effector cells to tolerate healthy cells of the organism, but also to ignore the non-self-commensals, such as the ones present in the intestine or on epidermis, as long as they do not induce tissue damage. However, once activated against a given antigen, the immune system shows a tendency of epitope spreading, which due to molecular mimicry could lead to cross-reactivity against bystander epitopes on healthy cells, especially under local proinflammatory conditions. Indeed, this phenomenon is thought to be responsible for a range of autoimmune disorders, including autoimmunity against skin antigens [4]. To prevent the bystander attack, the evolution has developed molecular mechanisms that tightly regulate the intracellular signal transduction following antigen recognition by T-cell receptor (TCR). These mechanisms have been commonly named “immune checkpoints”. To properly discriminate the effector functions of lymphocytes, there are two main types of immune checkpoints: Negative (inhibitory) and positive (stimulatory). mAbs targeting negative ICs are commonly known as “immune checkpoint inhibitors” (ICIs). 

The first molecule from the negative immune checkpoint family, cytotoxic T-lymphocyte-associated protein 4 (CTLA-4, CD152), was identified in 1987 [5] and its crucial role as a negative regulator of T cell activation was finally validated in 1995 [6,7]. CTLA-4 expression is dynamically upregulated in activated T cells and reaches the peak value between 24 and 48 hours following activation [8,9]. Once expressed, CTLA-4 potently competes with CD28 in binding to their cognate ligands, CD80 and CD86 [10], and thus strongly inhibits the priming phase of T cell activation [11]. CTLA-4 may also downregulate the intracellular signaling from the TCR complex via recruiting phosphatases [12], although the molecular events related to this phenomenon remain elusive. Subsequent studies by the 2018 Nobel Prize winner, Prof. James P. Allison, have demonstrated that, while indispensable for self-tolerance in the state of health, CTLA-4 is unfortunately also responsible for pathological tolerance towards malignant growth [13]. In consequence, monoclonal antibodies blocking CTLA-4 were shown to induce, in a “brake-off” mechanism, a potent antitumor response [13].

In parallel to studies on CTLA-4, the research group led by Prof. Tasuku Honjo, the second 2018 Nobel Prize awardee, has been focusing their studies on the Programmed Cell Death-1 (PD-1, CD279) receptor on immune cells [14]. PD-1 expression is also induced on activated T cells [15] and the cognate ligands for this molecule are PD-L1 and PD-L2, expressed on the target cells [16]. Therefore, the interaction between PD-L1/2 and PD-1 potently inhibits the effector phase of the cytotoxic immune response [17], which directly protects healthy tissues [18,19], but, when amplified in the tumor, also malignant cells [20]. Here again, monoclonal antibodies targeting either PD-1 or PD-L1 have been proved a powerful weapon against cancer [21], acting in the “brake-off” manner. 

**Figure 1:**
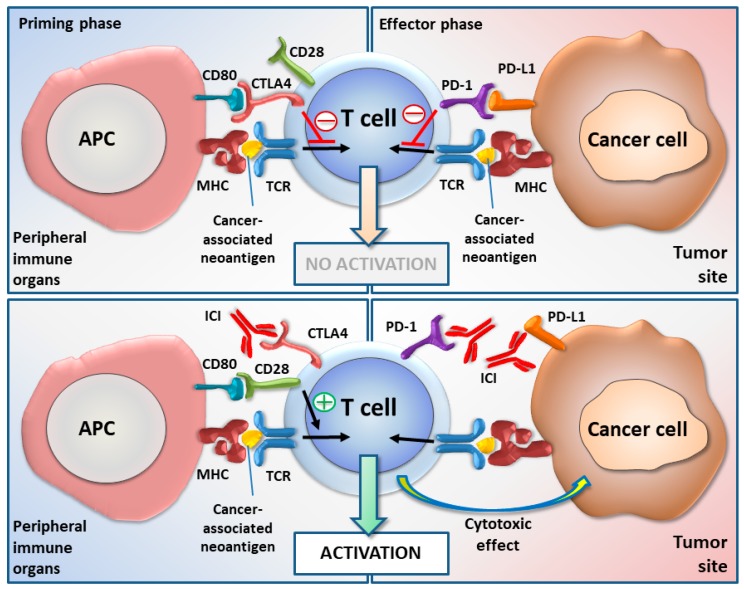
The mechanism of action of immune checkpoint inhibitors (ICIs).

At present, mAbs targeting CTLA-4 and PD-1/PD-L1 axis have an established place in oncology. Their mechanism of action is summarized in the Figure 1. It should be noted that while the expression of CTLA-4 ligands—CD80 and CD86 is limited to antigen presenting cells (APC), PD-L1 and PD-L2 can be expressed both by APCs and tumor cells. 

Following the discoveries of the roles of CTLA4 and PD-L1/PD-1 in the malignant growth, targeting CTLA-4, PD-1 or PD-L1 with five agents (ipilimumab, cemiplimab, nivolumab, pembrolizumab and avelumab) has been established as registered therapies in the treatment of cutaneous neoplasms (Table 1). Additionally, two other checkpoint inhibitors (durvalumab and atezolizumab) are potential agents to be applied in dermatooncology (Table 1).

Following the success of targeting CTLA-4, PD-1 or PD-L1, a range of other molecules have been proposed to act as negative immune checkpoints protecting cancer tissue, and also proposed attractive targets for therapeutic antibodies (reviewed in [22]). The examples of such molecules are: Lymphocyte-activation gene-3 (LAG-3), T-cell immunoglobulin and mucin-domain containing-3 (TIM-3), T cell immunoreceptor with Ig and ITIM domains (TIGIT), V-domain Ig suppressor of T cell activation (VISTA) or B7/H3 (CD276), and more molecules are joining this list [23]. mAbs against these novel targets are still being investigated in Phase 1/2 clinical trials.

## 2. Melanoma

Melanoma, the deadliest skin cancer, is responsible for the majority (approximately 75%) of deaths related to skin malignancies [24]. Melanoma formation is a result of a malignant transformation of melanocytes following the influence of both tumor-intrinsic and immune-related triggers [24,25]. The standard treatment for the localized disease consists of wide local excision with different safety margins [26]. Given a substantial recurrence risk after surgery, several attempts to develop adjuvant therapy have been made. Currently, the standard adjuvant therapy for recurrent locoregional disease includes anti-PD-1 nivolumab or pembrolizumab or dual BRAF-MEK inhibition by the combination of dabrafenib plus trametinib for *BRAF V600* mutated cases [24]. In metastatic disease, the combination of BRAF-MEK inhibitors is applied in *BRAF*-mutated patients, while the therapeutic options in unmutated patients have by now been scarce. In a small subset of patients with KIT kinase mutations, tyrosine kinase inhibitors have been tested with encouraging response rates characterized, however, by short duration [27]. Therefore, there was an urgent need for novel therapeutic option that is currently being fulfilled with the advent of checkpoint inhibitors.

### 2.1. Checkpoint Inhibitors in Melanoma Treatment

ICIs have an already established position in the treatment of melanoma. Melanoma is considered an immunogenic tumor due to a large number of somatic mutations mostly following ultraviolet damage from sun exposure. The subsequently produced tumor neoantigens are thus available for checkpoint inhibitor-reactivated immune cells [28]. The spontaneous regression of melanomas observed in some individuals is associated with functional T-cell activation [28]. Moreover, there is a body of evidence showing a link between impaired function of the immune system and melanoma proliferation [29]. Melanoma cells frequently evade the immune system by overexpressing negative immune checkpoints e.g. CTLA-4 [30]. PD-L1 can also be up-regulated following JAK-STAT activation [31]. Therefore, targeting ICs with mAbs have emerged as an attractive therapeutic option.

Initially, ICIs have been tested as adjuvants. The first trials concentrated on blocking CTLA-4 by ipilimumab. Encouraging results have been obtained in Phase III EORTC-18071 trial (NCT00636168) [32] on the adjuvant use of ipilimumab compared with placebo in patients with stage III melanoma, where a significant improvement in recurrence-free survival (RFS) has been achieved. Despite frequent adverse events (AEs), ipilimumab has been registered for patients with stage III melanoma at high risk of recurrence following complete resection in Oct 2015. The results of the EORTC 1325/Keynote-054 trial (NCT02362594) of pembrolizumab versus placebo in patients with stage III melanoma also demonstrated a significant improvement in RFS. Interestingly, a positive impact on RFS was noted in both PD-L1 positive and negative population and independently on the BRAF mutation status. [33]. Thus, pembrolizumab was approved as adjuvant therapy in stage III melanoma in Europe in Dec 2018 and by Food and Drug Administration (FDA) in Feb 2019. The superiority of checkpoint blockade with anti-CTLA-4 or anti-PD-1 mAbs to chemotherapy in the treatment of stage IV (metastatic) melanoma, has been proven in multiple randomized clinical trials (excellently reviewed in [24]).

The next step was the introduction of ICIs as a first-line therapy. Despite encouraging results of ipilimumab in terms of a marked increase in the percentage of long-term survival demonstrated in a pooled analysis from 12 clinical trials [34], due to its significant toxicity, anti-PD-1 nivolumab and pembrolizumab were further investigated. Given the positive impact on overall survival (OS) and low AE’s rate when compared to ipilimumab [35,36], both agents were approved in 2014 and are now a standard treatment in advanced melanoma according to The National Comprehensive Cancer Network (NCCN) guidelines. Following the results of pivotal Phase I CheckMate-069 (NCT01927419) and Phase III Checkmate-067 study (NCT01844505) testing the efficacy of nivolumab vs. nivolumab + ipilimumab [37,38], the combination of these two agents have been approved for the treatment of unresectable or metastatic melanoma, firstly in BRAF unmutated cases in Oct 2015, which has been further expanded independently on the BRAF mutation status in Jan 2016. However, due to substantial toxicity of the combination, a more intense monitoring is recommended in case of the combinational treatment [39]. Tremelimumab, another anti-CTLA4 monoclonal antibody, is also being examined. Despite promising results from the phase I study (NCT00257205) [40], a Phase III clinical trial (NCT01704287) of tremelimumab in comparison to the standard of care—dacarbazine—showed no significant benefits [41]. 

Currently, also other agents targeting the PD-1/PD-L1 axis are being tested in advanced melanoma:

- Cemiplimab—a small exploratory tumor biopsy-driven study to understand the relationship between biomarkers and clinical response in melanoma patients (NCT03002376)—ongoing.
- Avelumab:
  - Combination immunotherapy with vaccine in subjects with melanoma who have progressed on or after chemotherapy and PD-1/PD-L1 therapy (NCT03167177)—not yet recruiting.
  - In metastatic or locally advanced solid tumors (NCT01772004)—preliminary results show durable responses, promising survival outcomes and an acceptable safety profile in patients with previously treated metastatic melanoma [42].
  - In combination with other cancer immunotherapies in advanced malignancies (NCT02554812)—currently recruiting.
- Durvalumab:
  - Phase 1 safety and tolerability in combination with dabrafenib and trametinib or with trametinib alone (NCT02027961)—awaiting results’ publication.
- Atezolizumab—tested in numerous clinical trials.

### 2.2. Rituximab in Melanoma

Rituximab, a chimeric IgG1 kappa mAb targeting CD20 antigen, has an established use in the systemic treatment of B-cell malignancies. The interest in its use in melanoma is motivated by two different aspects of this disease. Firstly, CD20 has been identified as a marker of melanoma-initiating stem cells [43]. Regression of metastatic melanoma by targeting stem cells with rituximab has been described [44]. The second rationale for using rituximab is based on the concept of specific B-cell depletion [14]. Tumor-associated B-cells (TAB) have been identified in both primary and metastatic melanoma lesions [45,46,47] and the release of insulin-like growth factor by TABs leads to resistance to BRAF or MEK inhibitors [5]. By reducing the number of circulating B lymphocytes, rituximab might thus have a preventive effect on the development of drug resistance. However, the role of B cells in supporting the tumor growth and their possible use as a prognostic factor in melanoma patients are controversial [48,49,50,51], which is reflected by the conflicting results of rituximab’s use in preclinical studies and clinical observations in melanoma patients. On one hand, B cells have been described as a pro-tumorigenic population that dampens immune responses through secretion of anti-inflammatory cytokines such as IL-4, IL-10 or TGFβ. On the other hand, B cells are vital elements in shaping an effective immune response by being efficient antigen-presenting cells (APCs) for the expansion of tumor-associated antigen-specific CD8+ and CD4+ T cells (reviewed in [52]).

In syngeneic mouse models depletion of mature CD20+ B cells was shown to promote melanoma growth [53,54]. Moreover, B-cell depletion has been shown to increase the efficacy of therapeutic anti-melanoma vaccines in a syngeneic murine model [55]. The results from a small trial testing the efficacy of anti-CD20 ofatumumab in stage IV metastatic melanoma resistant to BRAFV600E inhibitors also further support the benefit of the anti-CD20 approach in melanoma [56]. 

Moreover, a case series of seven patients with metastatic melanoma treated individually with rituximab, suggested that anti-CD20 therapy might be a therapeutic option for metastatic melanoma [57]. Reports suggesting that rituximab administration may lead to melanoma induction or worsening are also known [58,59]. A delayed growth of melanoma in B cell-deficient mice has been reported for D5 melanoma cell line [60]. However, data from a large cohort analysis established no increased risk of melanoma following rituximab treatment [61].

Recently, TABs have emerged as predictors of resistance to ICIs. [54]. The analysis of datasets from anti-PD1-treated melanoma patients revealed increased B-cell numbers in pre-therapy tumor samples in patients responding to immune checkpoints therapy. 

### 2.3. Targeting LAG-3

Lymphocyte activation gene-3 (LAG-3, CD223) expressed on activated T, natural killer (NK) and B cells [62] is a negative regulatory protein for T cell function implicated in preventing tissue damage and autoimmunity [63,64]. In melanoma, LAG-3, frequently co-expressed with PD-1 was demonstrated on CD8+ tumor-infiltrating lymphocytes (TILs), leading to their clonal exhaustion and promotion of tumor growth [65]. An anti-LAG-3 mAb LAG525 is being tested in a phase 1/2 clinical trial in combination with an anti-PD-1 spartalizumab (NCT02460224). Another LAG-3 targeting mAb relatlimab (BMS-986016) alone and in combination with nivolumab is being tested in melanoma patients previously treated with anti-PD-1/PD-L1 therapy (NCT01968109). The preliminary results demonstrated encouraging initial efficacy with an objective response rate (ORR) of 16% and a disease control rate of 45% with benefit in some patients treated with anti-PD-1 [66].

### 2.4. Targeting TIGIT

TIGIT is a poliovirus receptor (PVR)-like protein containing Ig and ITIM domain with a well-established role in controlling immune suppression [67]. It is expressed on both T and NK cells, providing an opportunity to target the adaptive and innate arms of the immune system. TIGIT co-expression with PD-1 on CD8+ T cells was reported in melanoma [68,69] and its elevated expression correlates with poor prognosis. In in vitro studies, concomitant TIGIT and PD-1 blockade additively increased proliferation, cytokines production and degranulation of both tumor antigen-specific CD8+ T cells and CD8+ TILs from advanced melanoma patients [68]. Currently, three phase I clinical trials of different TIGIT-targeting molecules are opened in solid tumors, including melanoma, in combination with nivolumab—etiglimab (OMP-313M32)—NCT03119428, tiragolumab (MTIG7192A)—NCT02794571 and BMS-986207—NCT02913313.

### 2.5. Targeting TIM-3

T cell immunoglobulin-3 (TIM-3) detected on the surface of different types of immune cells is described as a direct negative regulator of T cells [70]. High TIM-3 levels on T cells are typical for exhaustion phenotype and correlate with poor prognosis in some tumors [64]. Interestingly, TIM-3 is also expressed by the melanoma cells themselves [71]. Data from the murine model showed promising results of TIM-3 targeting in combination with an anti-melanoma vaccine [72]. Since TIM-3 upregulation is associated with CD8+ T cells exhaustion in melanoma [73], clinical trials of two TIM-3 targeting mAbs—TSR-022 (NCT02817633) and MBG453 (as a single agent and in combination with PDR001—NCT02608268) are currently recruiting patients.

### 2.6. Targeting B7-H3

B7-H3 (CD276), a member of B7 family of immunoregulatory proteins expressed on dendritic cells, monocytes and macrophages is known to activate T-cells [74]. Its overexpression by melanoma cells and their microenvironment [75] results in immune escape and tumor proliferation [76,77]. Of note, B7-H3 shows limited expression in normal tissue, which may help to reduce the risk of adverse effects. Enoblituzumab (MGA271), a humanized anti-B7-H3 IgG1κ mAb, is being tested in combination with pembrolizumab in refractory cancer patients, including melanoma (NCT02475213). A phase I study of enoblituzumab in combination with ipilimumab in refractory melanoma (NCT02381314) has been completed and awaits the publication of the results. 

### 2.7. Targeting VEGF

VEGF inhibition seems a rational approach in melanoma therapy, as the progression of this tumor from radial to the vertical growth phases has been associated with increased microvessel density [78]. Besides promoting angiogenesis, VEGF impairs dendritic cell maturation and modulates lymphocyte endothelial trafficking [79]. Bevacizumab, a humanized IgG1 anti-VEGF mAb registered in the management of diverse solid tumors e.g., colorectal, lung and ovarian cancer, showed encouraging results in phase II clinical trials in combination with the cytotoxic alkylating agents fotemustine (NCT01069627) [80] and temozolomide (NCT00568048) [81]. A large phase III study (ISRCTN 81261306) tested adjuvant bevacizumab vs. observation in melanoma patients at high risk of recurrence. Despite a significant improvement in the disease-free interval, no significant differences in the OS between treatment and observation groups have been reported [82]. Bevacizumab is being tested in advanced metastatic melanoma in combination with immune checkpoint inhibitors—atezolizumab (NCT03175432), pembrolizumab (NCT02681549), nivolumab/avelumab (NCT03167177), ipilimumab (NCT00790010, NCT01950390 and (NCT0215852). Incidence of the immune-mediated tumor vasculopathy induced by ipilimumab [83] led to a non-randomized clinical study assessing the efficacy of dual CTLA-4 plus VEGF inhibition [79] that reported a disease-control rate (DCR) of 67.4% and beneficial safety profile providing a basis for further investigation.

### 2.8. Targeting Chondroitin Sulfate Proteoglycan 4 (CSPG4)

CSPG4, a transmembrane proteoglycan, is a highly immunogenic tumor antigen associated with melanoma formation and poor prognosis [84]. Multiple studies showed its role in the survival, growth and motility of melanoma cells in vitro and tumor formation in vivo (reviewed in [85]). Although it has been suggested as a potentially attractive target, no clinical trials addressing CSPG4 targeting have been proposed yet.

## 3. Basal Cell Carcinoma and Cutaneous Squamous Cell Carcinoma

Basal cell carcinoma (BCC) and cutaneous squamous cell carcinoma (CSCC) are both linked to UV exposure, chemical carcinogens, ionizing radiation and immunosuppression [86]. BCC is the most common skin malignancy among the white population [87]. Although it rarely metastasizes, it can be locally destructive. BCC is most commonly cured by local resection and in case of metastatic disease some novel drug modalities i.e., hedgehog inhibitors have recently emerged. However, there is a lack of further therapeutic options for progressing patients [88]. CSCC is more aggressive than BCC and accounts for up to 20% of all deaths from skin cancer [86]. Here, the lack of effective therapeutic options for recurrent and metastatic patients also poses a considerable problem. 

### 3.1. Checkpoint Inhibitors

In the treatment of metastatic BCC and CSCC there is also a large interest in ICIs supported by independent lines of evidence. First of all, early research in the murine models of UV-induced tumors showed immunosuppressive properties of the tumor microenvironment characterized by the onset of suppressor T cells [89]. High mutational burden present in BCC and CSCC, as a consequence of UV-exposure may elicit an effective immune response by inducing the expression of immunogenic tumor neoantigens [90]. This response can be further boosted by blocking the immunosuppressive checkpoint molecules. In fact, high mutation burden has been shown as a good response predictor to anti-PD-1 therapy in a panel of 12 different human tumors [91,92,93], where rapid expansion of neoantigen-specific T cell clones reactive to tumor neoantigens was observed. Data from the preclinical model of DMBA/PMA-induced carcinogenesis representing a multistage squamous cell carcinoma (SCC) development have also shown the efficacy of anti-PD-1 blockade in delaying the development of murine SCC [94].

There are several reports of response to the off-label treatment of BCC and CSCC with PD-1 targeting [95,96]. Other observations [97,98] have suggested that anti-PD-1 targeting may be a rational solution in BCC patients with acquired resistance to hedgehog pathway inhibition [97]. Substantial improvement in metastatic BCC with acquired resistance was noted after nivolumab [99] in five heavily pretreated patients with locally advanced and/or metastatic CSCC or baso-squamous carcinomas [100,101]. 

Pembrolizumab has been also tested in a small Phase II clinical trial (NCT02964559) in metastatic CSCC patients not curable by surgery or radiation. [102]. Data from a proof-of-principle, nonrandomized, open-label study of pembrolizumab with or without the hedgehog inhibitor vismodegib (NCT02690948) in patients with advanced BCCs showed no superiority of the combinational therapy vs. pembrolizumab alone and an acceptable safety profile [103].

A novel fully human anti-PD-1 monoclonal antibody—cemiplimab (formerly known as REGN2810)—is currently evaluated in BCC and CSCC (NCT02383212) [104]. The combined analysis of the phase 1 (NCT02383212) and phase 2 (NCT02760498) study in patients with locally advance or metastatic CSCC cemiplimab induced a response in approximately 50% of the patients [105]. The antibody received approval by US FDA in September 2018 for the treatment of patients with metastatic or locally advanced CSCC who are not candidates for curative surgery or curative radiation [106] and in April 2019 has been granted conditional marketing authorization in the EU for the same indication.

### 3.2. EGFR-Targeting 

Before the advent of the era of ICIs, there has been a large interest in the targeting of epidermal growth factor receptor (EGFR). EGFR, a member of the family of transmembrane tyrosine kinases plays a pivotal role in signal transduction pathways regulating proliferation, invasion and metastasis [107]. mAbs targeting EGFR—cetuximab and panitumumab—are clinically used in solid tumors. There are several case reports showing their potential use with an acceptable safety profile in unresectable or recurrent CSCC [108,109,110,111]. It has also been suggested that anti-EGFR may improve response rates in CSCC when combined with other targeted therapies [112,113]. However, since other therapeutic options emerged i.e., hedgehog inhibitors in BCC and ICIs in CSCC, no further trials for cetuximab were undertaken.

## 4. Merkel-Cell Carcinoma

Merkel-cell carcinoma (MCC) is a rare and highly aggressive skin cancer in most cases caused by the Merkel cell polyomavirus (MCPyV or MCV) [114]. As the infection with MCV is common, additional cellular events together with loss of immunosurveillance are postulated to contribute to MCC development [115]. Indeed, an increased incidence of MCC has been reported for cancer patients [116,117], patients with immune deficiencies e.g., HIV infection [118], transplant recipients treated with immunosuppressive agents [119] as well as patients with autoimmune diseases on immunosuppression [120]. Until recently, chemotherapy has been offered to patients with advanced MCC. However, limited efficacy of regimens based on cyclophosphamide, doxorubicin and vincristine was reported leading to the introduction of platinum agents in combination with etoposide, that still offer only a short-term response [121]. Thus, preclinical and clinical studies testing targeted therapies in MCC including ICIs are currently underway (reviewed in [122]).

### Checkpoint Inhibitors in MCC

Due to the immunogenicity of MCC [123] ICIs can be an effective approach in this malignancy. MCC-infiltrating CD8+ T cells, including MCPyV-specific T cells, have been shown to express high levels PD-1 and TIM-3, at far higher levels than T cells specific for other common human viruses [124]. Pembrolizumab was the first ICI to demonstrate objective tumor regressions in patients with MCC prompting its addition to the NCCN guidelines. A small single-arm, open-label Phase II clinical trial focused on treatment-naïve patients with stage IIIb or IV MCC (NCT02267603) showed a good tolerance [125]. Currently, the only registered ICI is avelumab that has been granted accelerated approval in Mar 2017 for the treatment of metastatic MCC, including chemotherapy-naïve individuals based on a multicenter Phase II clinical trial (NCT02155647) demonstrating a clinically meaningful and durable ORR [126]. The durability of responses to avelumab appears substantially superior comparing to historical trials of patients of chemotherapy [127,128,129]. The safety profiles of anti-PD-L1 antibodies administered to patients with MCC appear similar to those from previous trials involving patients with other tumor types. Combinational trials with avelumab are being considered e.g., with localized radiation or interferon-β (IFN-β) with or without adoptive immunotherapy of MCPyV T-antigen-specific T cells (NCT02584829) are currently ongoing. 

Nivolumab’s use has been investigated in the neoadjuvant setting for resectable MCC in a phase 1/2 trial (NCT02488759) [130]. Computed tomography scan results demonstrated tumor regression in 80% of patients.

Anti-CTLA-4 ipilimumab was also evaluated in an adjuvant trial following surgical resection of MCC (NCT02196961) [131]. However, after a median follow-up of 22.3 months, the enrollment to the study has been stopped due to the lack of efficacy of ipilimumab and a significantly increased incidence of adverse events. Another study aiming at assessing the combination of an anti-CTLA-4 tremelimumab with anti-PD-L1 durvalumab and polyinosinic-polycytidylic acid, and poly-L-lysine (polyICLC) (a TLR3 agonist; NCT02643303) is currently recruiting patients. [132]. Overall, these studies demonstrate the clear clinical benefit of immune checkpoint inhibition in MCC, which is superior to any form of therapy used hitherto. 

## 5. Primary Cutaneous Lymphomas

Primary cutaneous lymphomas (PCTs) are a heterogeneous group composed of cutaneous T-cell lymphomas (CTCLs) accounting for 75–80% of all PCTs, and cutaneous B-cell lymphomas (CBCLs) representing 20–25% of PCTs [133]. According to the WHO EORTC classification, they are defined as non-Hodgkin lymphomas present in the skin without the evidence of extracutaneous disease at the time of diagnosis [134]. 

### 5.1. CTCL

CTCL arises from the malignant proliferation of skin-homing CD4+ T cells [135] and is typically a disease of elderly people [136,137]. The main two subtypes of CTCL include the most frequent mycosis fungoides (MF), accounting for approx. 60% of CTCL, and a rare leukemic variant—Sézary syndrome (SS)—representing around 5% of CTCL. The second most common group, representing approx. 25% of CTCL, is primary cutaneous CD30+ lymphoproliferative disorders (LPDs) including primary cutaneous anaplastic large lymphoma (C-ALCL) and lymphomatoid papulosis (LyP) [133].

In the majority of cases of CTCL, early-stage MF is diagnosed and the disease can be managed with active observation or topical therapy using corticosteroids, chemotherapy (mechlorethamine), immunomodulators (imiquimod), radiation or phototherapy [136]. The 5-year survival for these patients is around 90% compared with 30–50% for advanced disease [138]. Prognosis in SS is poor with an overall treatment response rates varying from 7.5 to 22.4 months [139]. Besides standard therapy (e.g., extracorporeal photopheresis (ECP), photochemotherapy, retinoids, radiation therapy, IFN-α, low dose methotrexate and polychemotherapy), small-molecule inhibitors and mAbs are currently being explored in this malignancy [140,141]. All those treatment modalities have, however, relatively low response rates ranging from 14% to 60% (mostly 20–30%) and median duration of response rarely exceeding 1 year [142]. Therefore, given the fact that allogeneic stem cell transplantation is the only curative option by now, there is a need for novel therapies in CTCL. 

#### 5.1.1. Alemtuzumab

Alemtuzumab, a humanized IgG1 kappa mAb targeting CD52 antigen expressed on both benign and malignant B and T cells, monocytes, macrophages, natural killer cells and a proportion of granulocytes [143] has originally been approved for the treatment of chronic lymphocytic leukemia (CLL). Given the expression of CD52 antigen on CD4+ T cells, there has been an increasing interest in applying alemtuzumab in CTCL. Alemtuzumab showed efficacy in managing erythroderma, plaque or skin tumors and importantly reduced pruritus. Results from a multicenter retrospective analysis carried among 39 patients with advanced CTCL (23 with SS and 16 with advanced MF) treated with alemtuzumab i.v. showed a 70% ORR in patients with SS and 25% ORR in patients with MF. The reason for its inefficacy in MF lies in the different origin of malignant T cells than in SS [144]. A presence of diffuse erythema has been suggested as a predictor of complete and durable response [145]. However, alemtuzumab increased the risk of infection due to depletion of B and T cells leading to cytomegalovirus (CMV) reactivation, fever of unknown origin and generalized herpes simplex infection. In the last years three clinical trials assessing alemtuzumab’s efficacy in MF and SS have been completed (NCT00057967, NCT00047060 and NCT00057967), however their results have not been published so far. 

#### 5.1.2. Brentuximab Vedotin

Brentuximab vedotin (BV, formerly known as SGN-35) is an antibody-drug conjugate consisting of a CD30-directed monoclonal chimeric IgG1 antibody and monomethyl auristatin E (MMAE), a microtubule disrupting agent. CD30 is an excellent target for immunotherapies, both for mAb and chimeric receptor (CAR) lymphocytes due to its limited expression on non-malignant immune cells [146]. The mechanism of action of BV consists of the internalization of the drug-conjugate after binding to CD30-expressing cells, followed by the release of MMAE leading to cell cycle arrest and apoptosis [147,148]. BV, originally registered for the treatment of Hodgkin lymphoma and primary cutaneous large cell anaplastic T cell lymphoma (pcALCL), based on the results of phase III trial (NCT01578499) in Nov 2017 gained approval for CTCL patients who have received prior systemic therapy [149,150]. Another clinical trial of BV in CD30-positive ALCL, MF and LyP (NCT01352520) is ongoing with encouraging results in LyP and MF [151,152]. Due to the incidence of peripheral neuropathy, protocols with lower dosage have been suggested. The mAb also shows potential in the treatment of rare primary cutaneous natural killer/T-cell lymphoma with aberrant CD30 expression [153,154]. 

#### 5.1.3. Mogamulizumab 

Mogamulizumab, a humanized afucosylated IgG1 targeting CC chemokine receptor type 4 (CCR4) is the second mAb registered for the management of CTCL. CCR4, involved in cell trafficking of lymphocytes to the skin, is consistently expressed on the surface of tumor cells in T-cell malignancies [155,156]. Defucosylation of the mAb results in an increased affinity to FcγRIIIa (CD16), and enhanced antibody-dependent cell-mediated cytotoxicity (ADCC) [157]. It has been approved in Aug 2018 for the patients with relapsed or refractory CTCL after at least one prior systemic therapy. The registrations were based on the results of Phase III clinical trial (NCT01728805) including patients with relapsed MF or SS on either mogamulizumab or vorinostat. Median PFS for mogamulizumab reached 7.6 months and the ORR 28% compared to 3.1 months and 5% for vorinostat [158]. Moreover, in patients treated with mogamulizumab an improvement in some aspects of quality of life, including skin pain and fatigue were reported. Interestingly, the clinical response to mogamulizumab was not associated with skin CCR4 expression. [159]. Despite the relatively good safety profile of the antibody in clinical trials, some rare serious adverse events with potentially fatal outcome have been reported in clinical experience, mostly in patients with adult T-cell leukemia lymphoma (ATLL). [160,161]. By targeting CCR-4 mogamulizumab also eliminates nonmalignant regulatory T cells (Tregs) leading to autoimmune disorders [162] and predisposing to increased risk of graft-vs.-host disease (GVHD) after allogeneic bone marrow transplantation [163,164,165,166]. Therefore, in mogamulizumab-treated patients transplantation should be delayed for at least 50 days from the last dose and a Treg count prior to transplant has been suggested [167].

#### 5.1.4. KIR3DL2 Targeting

KIR3DL2, also known as CD158k, a member of the killer cell immunoglobulin-like receptor (KIR) family, was initially identified as an inhibitory co-receptor on the surface of NK cells. In healthy individuals, KIR3DL2 is expressed by about 20% of NK cells and also by a small proportion of CD4^+^ (5%) and CD8^+^ (9%) T cells [168]. KIR3DL2 overexpression by MF and SS cells correlates with the disease stage and large cell transformation [169], as well as shorter survival [170]. Therefore, targeting KIR3DL2 raises hopes for developing the long-awaited CTCL-targeted therapy [171]. IPH4102, an anti-KIR2DL2 humanized IgG1 mAb effectively induces ADCC and immunophagocytosis [172], delays tumor growth and improves the overall survival in a xenograft mouse model. The results of a phase I study in MF and SS patients (NCT02593045) demonstrated a confirmed global overall response in 16 of 44 patients, and of those, 15 responses were observed in 35 patients with Sézary syndrome [173]. Moreover, a phase II trial of IPH4102 alone or in combination with chemotherapy in patients with advanced T cell lymphoma (NCT03902184) is currently recruiting patients.

#### 5.1.5. Immune Checkpoint Inhibitors 

Although the data on the expression pattern of PD-1 axis in CTCL are limited, they suggest a potential role of these negative immune regulators in the pathogenesis of CTCL and designate them as therapeutic targets. In general, these studies suggest a higher expression of PD-1 on CD4+ malignant cells in case of blood and skin of SS patients comparing to MF patients [174,175] and PD-1 has been proposed as a factor responsible for drug resistance in SS [176]. Our unpublished observations also indicate higher levels of PD-1 expression on CTLA + CD4 + cells in patients with a higher tumor burden. The particularity of CTCL in the context of the implementation of immune checkpoint inhibitors relies on the fact that the tumor itself arises from CD4+ T cells, a population of lymphocytes responsible for the priming of cytotoxic response. In CTCL both malignant and bystander T helper cells are characterized by Th2 bias, that results in skewed anti-tumor response [177]. A mounting body of evidence suggests that in CTCL both CD4+ and CD8+ cells have characteristics of immune exhaustion [178,179,180]. Therefore targeting immune checkpoints would have implications on the functionality of both helper and cytotoxic T cells. By now, the role of PD-1 axis has been much more investigated in CD8+ T cells [181]. It is not yet clear how targeting this pathway affects Th2 phenotype in CTCL. Studies in solid tumors suggest that blocking PD-1 may be effective in abrogating Th2 bias. PD-1 blockade was found to shift antigen-induced cellular reactivity toward a proinflammatory response, enhanced production of interferon- γ (IFN-γ), IL-2, TNF-α, IL-6 and reduced production of anti-inflammatory cytokines IL-5 and IL-13 [182]. In ex vivo studies, the inhibition of PD-1 downstream signaling increased IFN-γ secretion in a subset of patients, suggesting that PD-1 targeting may abolish suppressive phenotype of SS cells [174]. In CTCL by now promising results have been reported in phase II trial of pembrolizumab (NCT02243579) in heavily pretreated advanced-stage MF and SS patients [183,184]. Combination studies based on pembrolizumab are warranted in order to increase response rates.

Currently five open-label multicenter clinical studies of anti-PD-1 pembrolizumab in monotherapy and in combinations are ongoing and one trial for nivolumab is recruiting patients. However, as PD-1 has recently been demonstrated to act as a tumor suppressor in T-cell malignancies [185], it has been discussed that targeting PD-1 ligands may potentially offer a safer option in CTCL. 

A study by Wilcox et al. demonstrated PD-L1 expression in peripheral blood CD4+ T cells in the majority of patients with leukemic CTCL, however only in 27% of patients’ biopsies as evaluated with immunohistochemistry [186]. Its expression, was high in the tumor environment, particularly in the monocyte-derived compartment, where PD-L1 was expressed by 73% of cells. Currently, one trial of anti-PD-L1 atezolizumab (NCT03357224) in relapsed or refractory CTLC is active and another one for durvalumab (NCT03011814) is recruiting patients.

While the PD-1/PD-L1 has gained much interest in the therapy of CTCL, not much is known about CTLA-4 expression in this malignancy. Increased CTLA-4 expression has been reported on the surface of CD3+ T cells from MF patients, most prominently in patients with late-stage disease [187]. In an analysis of skin samples, Querfeld et al. observed higher expression of CTLA-4 on both CD4+ and CD8+ T cells. In our study, we observed no significant differences in CTLA-4 expression in CD4+ malignant vs. by-stander T cells in SS patients and healthy controls [188]. There is only a limited amount of data reports on the use of anti-CTLA-4 in CTCL. In a phase I clinical trial the combination of nivolumab and ipilimumab displayed a similar clinical safety and efficacy when compared with nivolumab monotherapy among pretreated patients with CTCL and peripheral T-cell lymphoma [189]. Currently, there are no active or recruiting clinical studies testing the efficacy of CTLA-4 targeting in CTCL.

#### 5.1.6. CD47

CD47 is a principal ligand for signal regulatory protein alpha (SIRPα)—an immunoreceptor—expressed on all myeloid cells [190]. Binding to SIRPα results in a ‘do not eat me’ signal and suppresses phagocytosis by macrophages. Upregulation of CD47 by various types of cancer cells is one of the strategies of immunoevasion [191,192] contributing to poor prognosis for the patients [193,194]. CD47 is highly expressed on Sézary cells in the peripheral blood and skin and correlates with worse OS [195]. TTI-621, a recombinant fusion protein composed of human SIRPα N-terminal domain fused to the Fc receptor of IgG1 [196] not only blocks CD47’s ‘do not eat me’ signal but also enhances phagocytosis of tumor cells by monocytes. By now, it is being tested in two Phase I trials. A single intratumoral injection resulted in a marked decrease in tumor size and in the number of circulating SS cells in MF and SS patients (NCT02890368). Moreover, upregulation of IFN-associated genes and alterations in innate immunity activation genes in peripheral blood and tumor tissue have been observed [197]. Due to these encouraging results, further trials of TTI-621 in the management of CTCL are warranted [198].

### 5.2. CBCL

According to the WHO EORTC joint classification B-cell-derived PCLs are classified into three major subtypes: The most common primary cutaneous follicle-center lymphoma (PCFCL), primary cutaneous marginal zone lymphoma (PCMZL) and the rarest but aggressive primary cutaneous diffuse large B-cell lymphoma, leg type (PCLBCL, LT) [199]. Both PCMZL and PCFCL types are characterized by an excellent prognosis, with 5-year survival rates higher than 90%, while for PCLBCL the 5-year survival rate is lower than 60% [200]. Due to the lack of randomized controlled trials in CBCL, the treatment recommendations of EORTC and International Society for Cutaneous Lymphoma (ISCL) are mostly based on small retrospective studies and institutional experience [201,202]. Patients with PCMZL and PCFCL are mostly treated with low-dose radiation therapy, while PCLBCL is managed with rituximab combined with chemotherapy with or without radiotherapy [200]. Anti-CD20 therapy is employed in PCFCL and PCMZL with more widespread skin involvement. 

#### Rituximab

Rituximab applied intravenously is considered an alternative to the conventional treatment of PCFCL and PCMZL and is used in the treatment of PCLBCL, LT mainly in combination with cyclophosphamide, hydroxydaunorubicin [doxorubicin], Oncovin [vincristine], prednisone (CHOP) chemotherapy (R-CHOP). The treatment of PCDLBCL-LT is extrapolated from diffuse large B-cell lymphoma (DLBCL) due to morphologic, phenotypical and molecular genetic features as well as a clinical behavior they share [201,203]. However, it has to be noted that there is a lack of randomized clinical trials on this topic. Moreover, there are case reports on the use of rituximab as a monotherapy in PCLBCL, LT (reviewed in [204]) 

Data on the i.v. use of RTX in indolent CBCL are also case reports (reviewed in [204]). Intralesional application of RTX has been suggested as an alternative due to a better tolerance of treatment (the most common AE being injection-site pain) [205,206], lower dose and increased convenience for the patient with a slightly better outcome [201]. 

## 6. Problems with the Use of Checkpoint Inhibitors

In this part we will concentrate on discussing the problems concerning the clinical use of ICIs mainly in the context of melanoma. 

### 6.1. Resistance to Immune Checkpoints

The application of ICIs encounters the problem of both primary (innate) as well as secondary (acquired) resistance. Three populations of patients—(1) responders, (2) patients with innate and (3) those with acquired resistance—have been identified by analyzing the results of the clinical trials [207,208,209].

In the case of anti-PD-1 targeting, the incidence of primary resistance has been reported in approximately 40% of untreated patients, 65% of the patients after progression on other therapies and in >70% of those treated with ipilimumab [209]. The primary insensitivity to immune checkpoints is related to insufficient T-cell and macrophage infiltration of the tumor, lack of PD-1 expression in the tissue, inadequate amount of neoantigens and low mutational burden. Moreover, immunosuppressive factors within the tumor microenvironment i.e., the presence of an innate anti-PD-1 resistance signature (IPRES) transcriptional signature (27), the absence of an interferon signature, increase in Tregs number, upregulation of PD-L1 molecule and the induction of indoleamine 2,3-dioxygenase (IDO) [209,210] also play an important role. Additionally, numerous studies aim at defining the role of the gut microbiome in the responsiveness to immunotherapy [211]. These studies are motivated by the fact that unmethylated CpG oligodeoxynucleotides abundantly present in the bacterial DNA enhance CD8+ T cell anti-tumor immunity by downregulating PD-1 expression via the IL-12 pathway. Moreover, the observations in germ-free animal models showed reduced numbers and impaired function of DCs and macrophages. Indeed, a higher diversity of the gut microbiome correlates to an increased response to anti-PD-1 monotherapy [212]. Overall, the results from preclinical in vivo studies of fecal transfer in a murine model of melanoma [213] and sequencing of the gut microbiota composition from 42 melanoma patients treated with anti-PD-1 [214] suggest a correlation between the presence of *Bifidobacterium* species and clinical response to this immunotherapy. In patients treated with ipilimumab specific bacteria genera i.e., *Faecalibacterium* and *Bacteroides* [215,216] were also associated with clinical response. Other studies (reviewed in [209,217]) suggest the influence of other bacterial species clearly indicating that further studies on this topic are warranted.

The secondary resistance concerns approximately 30% to 40% of patients showing an initial response to anti-PD-1. Although the mechanisms underlying the acquired resistance are not completely deciphered it seems that the upregulation of alternative immune checkpoints i.e., TIM-3 and LAG-3 [218], *JAK2* mutations resulting in disrupted IFN-γ [219] and decreased expression of human leukocyte antigen (HLA) molecules leading to decreased antigen presentation [220] play a role (reviewed in [209]). 

Based on the finding from a retrospective study comparing the efficacy of ipilimumab monotherapy vs. ipilimumab + nivolumab in patients after progression on PD-1 inhibitors, ipilimumab seems an option for patients with acquired resistance [221]. Preclinical data from murine model suggest the efficacy of dual targeting of PD-1 together with the emerging immune checkpoints—LAG-3 [222] or TIM-3 [223]. 

### 6.2. Response Markers for Checkpoints Inhibitors

There is an unmet need for biomarkers that will identify patients more likely to respond to ICIs. The advances in the topic have been excellently reviewed in [224,225]. Here we aimed at accentuating the key aspects. As the blockage of PD-1/PD-L1 axis represents the most widely used ICI-based therapy, the majority of the cited studies concentrates on this aspect.

Data from clinical trials and cohort studies suggest that PD-L1 expression on tumor cells can be used as a predictor of response [226,227,228,229,230,231]. The expression of PD-L1 varies significantly depending on the melanoma subtype, which correlates with response to therapy [232]. However, its application as a single prediction marker of the therapy outcome has some limitation. Its expression undergoes dynamic changes in the course of treatment and as a result of inflammation [233,234] and there are reports on successful clinical outcome of anti-PD-1 treatment in PD-L1 negative cases [235]. Interestingly in Merkel cell carcinoma response has been observed independently on PD-L1 status [125]. PD-L1 expression in the tumor microenvironment has also been suggested to be more informative than its expression on the tumor cells [236,237]. Recently, soluble [238] and exosomal PD-L1 [239] have been presented as a possible predictor for anti-PD-1 therapy. High levels of circulating PD-L1 would suggest the exhaustion of T cells and the impossibility of their further reinvigoration following anti-PD-1 therapy. Interestingly, however, considerable changes in the levels of circulating PD-L1 prior to and during pembrolizumab [239] and ipilimumab [238] treatment have been observed and shown to correlate with clinical response. Some easily analyzable biochemical parameters have been suggested as potential response predictors e.g., lactate dehydrogenase (LDH) and S100, which are normally used as indicators of disease progression [240]. However, as all these markers do not correlate with the duration of response, they may identify patients with very high tumor burden that are unlikely to benefit from immunotherapies, but cannot be used as response predictors [225]. The same applies to the number of the organs involved by the tumor [241].

In terms of demographic factors it has been shown that although men are highly more susceptible to different types of tumors and have two-times higher risk of mortality from all cancers than women do [242], their relative survival benefit from ICI-based therapy is consistently higher than for women. Interestingly, the response to PD-1 blockage increases with age [243]. Paradoxically, despite the clear association between increased body-mass index (BMI) and the risk of developing and dying from various types of cancer [244], in a large retrospective study including a total of 2046 patients with metastatic melanoma obesity has been shown to increase response to all targeted therapies, including ICIs [245].

Features of the tumor microenvironment (TME) and the composition of the immune populations in the peripheral blood also associate with the response. Specifically, baseline levels of CD8+ TILs correlate with the likelihood of response. Moreover, the number of CD8+ TILs increases during therapy in responders [246], suggesting that the preexisting immunity is required for the ICI therapy efficacy. By analyzing the transcriptomes of 16,291 individual immune cells from 48 tumor samples of melanoma patients treated with ICI, two distinct states of CD8+ T cells associated with tumor regression or progression were defined by clustering [247]. The presence of the TCF7 transcription factor in CD8+ T cells that is crucial for their differentiation, self-renewal and reinvigoration has been presented to predict clinical response to checkpoint therapy. Since CD4+ T helper cells also play an important role in the tumor elimination e.g., by increasing the cytotoxic function of CD8+ cells and secretion of IL-2, several studies investigated them as a potential prognostic factor. An increase in central memory CD4+ T cells [248] and IL-9-producing CD4+ Th9 cells [249] has been reported exclusively in long-term responders to anti-PD-1 blockage. The recent advances in understanding the role of circulating myeloid-derived suppressor cells (MDSCs) in cancer progression also suggest that this population may play a role in defining survival and response to treatment [250]. Results from the studies in anti-PD-1-treated patients clearly show that a high percentage of MDSCs correlate with poor response to the therapy [251,252]. Further, the frequency of CD14+CD16-HLA-DRhi monocytes has been reported to predict progression-free and overall survival in response to anti-PD-1 immunotherapy [253]. Recently, it has been shown that antibodies specific for melanocyte differentiation antigens (MDAs) and cancer-testis antigens may be a response predictor for ICIs [254], suggesting also the importance of the interaction between B and T cells for the therapy outcome. 

By now, it seems that tumor mutational burden together with T cell-inflamed gene expression profile exhibit the greatest predictive utility in identifying responders and nonresponders to anti PD-1 therapy, as shown in a large study in >300 patient samples with advanced solid tumors and melanoma (representing approx. 30% of the tested samples) from four KEYNOTE clinical trials [255]. Large-scale analyses in various cancers including patients with basal cell carcinoma and melanoma univocally reported that patients with intermediate to high mutational tumor burden assessed with next-generation sequencing (NGS) show a better clinical response to the PD-1/PD-L1 blockade [93,255,256]. The number of mutations undergoes a marked decrease in immunotherapy-responders, as reported by a study in 68 advanced melanoma patients treated with nivolumab. As demonstrated by a clonality analysis, the tumors in patients with Complete Response/Partial Response (CR/PR) undergo a substantial evolution in the course of the therapy, already after four weeks from the therapy start [228,234]. Upregulation of other immune checkpoints such as LAG-3 or TIM-3 was reported among others. Notably, these molecules remain candidates for co-targeting in combination treatment regimens in order to boost immunotherapy efficacy.

### 6.3. Managing Adverse Events

Since immune checkpoints under physiological conditions regulate the immune response to prevent autoimmunity bystander damage of the normal tissue, their inhibition leads to a plethora of immune-related AEs (irAEs). Moreover, exacerbation of already pre-existing conditions [257,258] is reported in ICIs-treated patients. The incidence of irAEs in patients treated with ICs and methods of management have been reviewed in [259,260]. The guidelines on the management of AEs have been prepared by several organizations [261,262].

Fortunately, fatal AEs, with their frequency of between 0.3% and 1.3% are less frequent than in case of other therapeutics [263]. However, they occur much earlier in the course of treatment than in the case of other therapies and evolve rapidly [264]. Data from metanalyses indicate that in case of ipilimumab-treatment the most frequent cause of death is colitis (approx. 70%), while targeting the PD-1/PD-L1 axis is most frequently associated with fatal pneumonitis (approx. 35%), hepatitis (approx. 20%) and neurotoxicity (approx. 15%).

In general the irAEs can affect every organ. However, some differences regarding the organ distribution can be identified in the populations of patients treated with anti-CTLA-4 or anti-PD-1/PD-L1. While both these populations suffer from colitis, endocrinopathies and cutaneous toxicity, pneumonitis and nephritis are typical for the group treated with anti-PD-1/PD-L1 [259]. Skin toxicities following immune checkpoint inhibition in melanoma have been thoroughly reviewed in [265] and are mostly observed in ipilimumab-treated patients. Skin-related side effects, experienced by around 60% of patients, are rarely severe and are mostly limited to rash and itching occurring at the beginning of the treatment, with its peak at the sixth week [266]. Targeting of PD-1 drugs induces less skin toxicity than ipilimumab with the incidence of some form of skin disorders around 40% [267,268]. Based on the by now the most comprehensive study by Hwang et al. the most frequent are rashes that can be divided into lichenoid reactions (17%) and eczema (17%) and the third most common adverse reaction, vitiligo (12%) [269]. A rash can be treated symptomatically with emollients, topical corticosteroids, oral antihistamines and oral corticosteroids in exacerbated cases. Vitiligo can be managed by the use of topical corticosteroids that induce repigmentation and the use of broad-spectrum photoprotection is highly required. Other forms of skin toxicities include severe pruritus, psoriasiform reactions, widespread erythema, DRESS syndrome, photosensitivity, sensitivity/skin toxicity in previously irradiated areas, ulcerations pyoderma gangrenosum-like, acneiform rash, eruptive keratoacanthomas, Sweet syndrome, Grover´s disease, Stevens–Johnson syndrome/toxic epidermal necrolysis and erythema nodosum-like panniculitis.

Some authors suggest that cutaneous immune-related AEs of ICIs i.e., vitiligo and rash may be used as predictors of the clinical response. Indeed, analyses of nivolumab-treated melanoma patients indicate that the incidence of vitiligo and rash correlates with a significant OS improvement [270,271]. Other reports suggest that anti-PD-1-induced vitiligo associated with other toxicities, such as lichenoid reactions and eczema, can be a good prognosis marker [272].

## 7. Improving the Efficacy of ICIs

Beside strategies combining ICIs with other anti-tumor strategies i.e., conventional chemotherapy (reviewed in [273]), small-molecule inhibitors (reviewed in [274]), anti-angiogenic drugs and oncolytic viruses (both reviewed in [275]) and the already mentioned dual inhibition of immune checkpoints e.g., PD-1 and TIGIT, the efficacy of ICIs can also be improved by modulating the affinity of mAb to Fc receptors. Glyco-modification of the Fc portion of the antibody is routinely applied to eliminate ADCC induction in the case of two anti-PD-L1 IgG1 mAbs (atezolizumab and durvalumab) in order to avoid elimination of PD-1/PD-L1-expressing TILs [276]. Indeed, all anti-PD-1 directed mAbs belong to the IgG4 isotype with silenced ADCC activity. However, the utility of eliminating ADCC has been largely questioned by the efficacy of anti-PD-L1 avelumab [126] with non-suppressed ADCC activity. As the results of the studies in a murine tumor model strongly suggest that the activity of agents targeting PD-L1 relies on their binding to activating FcγR resulting in the altering myeloid subsets within the tumor microenvironment [277]. Recently, a defucosylated anti-PD-L1 mAb with increased affinity for FcγRIIIa has been engineered on the basis of atezolizumab and demonstrated encouraging in vitro results with increased CD8 T cell activation [278]. Therefore, glyco-optimization of the structures of ICIs may be of potential clinical benefit [279]. The anti-tumor efficacy dependent on the Fc-mediated effector functions has also been demonstrated for CTLA-4, TIGIT and VISTA (reviewed in [279]). However, this strategy is target-dependent, as the presence of FcγR-binding capacity compromises the anti-tumor activity of anti PD-1 mAbs [277]. In case of anti-PD-1 mAbs the attenuation of ADCC relies on the application of the IgG4 isotype. However, this isotype is considered anti-inflammatory and may result in a reduced anti-tumor efficacy, as it retains the binding to FcγRIIb [277]. Therefore, the efficacy of anti-PD-1 therapy may benefit from the development of agents with null FcγR-binding. It applies also to mAbs targeting co-inhibitory receptors i.e., TIM-3 and LAG-3 [279].

## 8. Conclusions and Perspectives

Skin is the human body’s largest organ, and its easy accessibility offers an unprecedented gateway for early detection and diagnosis of diseases. Both, solid and hematological malignancies can arise in skin or secondarily affect it. The incidence of skin cancers has been increasing over the past decades up to 287,000 new cases for melanoma and 1.04 million new cases for non-melanoma in 2018 worldwide. This makes skin cancers a global health burden and a field of intense research contributing to the advancement of treatments in oncology overall. Increased knowledge about signaling and immune pathways led to the development of targeted therapies and immunotherapies representing recent breakthroughs in cancer therapy.

These therapies opened the way for personalized and precise treatment strategies, but they also confront physicians with novel adverse drug reactions, with the skin being again among the most commonly affected organs.

In light of the above, further research should aim to identify more antibody-targetable molecules, accumulate data on how to predict response to treatment and manage the adverse events. In particular, identifying novel molecular targets that may provide a solution to decreased efficacy of mAbs due to antigen loss as a result of selective pressure is of the utmost importance. While the intracellular molecules represent nearly half of the human proteome and provide an immense reservoir of potential novel targets, they have not yet been extensively explored in oncology [280]. Targeting intracellular molecules aims also at exploiting the products of their degradation by the proteasome that are subsequently presented in the context of MHC class I molecules and recognized by CD8+ T cells. Antibodies targeting such MHC-peptide complexes, the so-called T-cell receptor-mimic (TCRm) antibodies expand the range of potential targets of immunotherapy without the problem with delivery, which is typical for intracellular antibodies [281]. Similar to conventional mAbs, TCRm antibodies activate various immune-dependent mechanisms i.e., ADCC and complement-dependent cytotoxicity (CDC). Such agents are being designed for melanoma treatment, however none of them has yet entered the clinic [280]. The use of bispecific mAbs also offers potential benefits in the context of the immune-rich skin microenvironment. An interesting example of the use of bispecific mAbs has been recently applied in in vitro studies, where a bispecific mAb targeting PD-L1xCSPG4 showed efficacy in the treatment of mixed cultures containing primary patient-derived CSPG4-expressing melanoma cells and autologous tumor-infiltrating lymphocytes [282]. Such bispecific mAbs may reduce the off-target binding to PD-L1-expressing normal cells that compromises on-target effect and is implicated in autoimmune-related (AEs).

## Figures and Tables

**Table 1:** Monoclonal antibodies (mAbs) targeting Cytotoxic T-Lymphocyte-Associated protein 4 (CTLA-4) and Programmed Cell Death-1 (PD-1)/PD-1 ligand-1 (PD-L1) currently in use in dermatooncology.

Name of the Agent	Molecular Target	Isotype	Source	Mechanism of Action	Trade Name	Registration Status in Dermatooncology	Most Relevant Clinical Trials
Ipilimumab	CTLA-4	IgG1	human	Triggers ADCC Blocks inhibitory APC—T cell interaction	Yervoy	2011—melanoma	NCT00636168 in MM, NCT01844505 in MM
Tremelimumab (CP-675206)	CTLA-4	IgG2	human	Reduced ADCC activity Blocks inhibitory APC—T cell interaction	NA	In clinical trials in melanoma	NCT00257205 in MM, NCT01704287 in MM
Cemiplimab REGN2810 (SAR439684)	PD-1	IgG4	human	Reduced ADCC activity Blocks inhibitory APC—T cell as well as tumor cell–T cell interaction	Libtayo	2018—CSCC	NCT03002376 in MM, NCT02383212 in BCC and CSCC, NCT02760498 in CSCC
Nivolumab	PD-1	IgG4	human	Reduced ADCC activity Blocks inhibitory APC—T cell as well as tumor cell–T cell interaction	Opdivo	2014—melanoma	NCT01844505 in MM, NCT01927419 in MM
Pembrolizumab (initially known as lambrolizumab)	PD-1	IgG4	humanized	Reduced ADCC activity Blocks inhibitory APC—T cell as well as tumor cell–T cell interaction	Keytruda	2014—melanoma	NCT02362594 in MM, NCT02243579 in CTLC
Atezolizumab	PD-L1	IgG1	humanized	Reduced ADCC activity Blocks inhibitory APC—T cell as well as tumor cell–T cell interaction	Tecentriq	In clinical trials in CTCL and melanoma	NCT03357224 in CTCL, NCT04020809 in MM, NCT01656642 in MM
Avelumab	PD-L1	IgG1	human	Triggers ADCC Blocks inhibitory APC—T cell as well as tumor cell–T cell interaction	Bavencio	2017—MCC	NCT01772004 in MM, NCT02155647 in MCC
Durvalumab (MEDI4736)	PD-L1	IgG1	human	Reduced ADCC activity Blocks inhibitory APC—T cell as well as tumor cell–T cell interaction	Imfinzi	In clinical trials in CTCL, melanoma and MCC	NCT02027961 in MM, NCT02643303 in CTCL

ADCC—antibody-dependent cell-mediated cytotoxicity, APC—antigen presenting cell, CSCC—cutaneous squamous cell carcinoma, CTCL—cutaneous T-cell lymphoma, NA—non-applicable, MCC—Merkel-cell carcinoma, MM—malignant melanoma.
